# Dr. William S. Cheesman

**Published:** 1912-06

**Authors:** 


Dr. William S. Cheesman of Auburn, died May 7, 1912, aged 60 years. Born in Brooklyn, February 10, 1853, he prepared for college in the Brooklyn Polytechnic Institute and entered Princeton in 1871. He graduated in 1875, and four years later graduated from the Columbia College of Physicians and Surgeons, at the head of his class. He located in Auburn in 1881 and resided there continuously.

He was a member of the American Medical Association, the Medical Societies of the State of New York, Cayuga county and city of Auburn. At one time he was president of the Central New York Medical Association, a member of the Syracuse Academy of Medicine, Fellow of the New York Academy of Medicine, and had recently been appointed to the Committee on Experimental Medicine, of the New York State Medical Society. His contributions to medical publications were numerous and meritorious. The deceased had been ill for a long time with nervous complications.

